# Edgetic Perturbations Contribute to Phenotypic Variability in PEX26 Deficiency

**DOI:** 10.3389/fgene.2021.726174

**Published:** 2021-11-04

**Authors:** Amelie S. Lotz-Havla, Mathias Woidy, Philipp Guder, Jessica Schmiesing, Ralf Erdmann, Hans R. Waterham, Ania C. Muntau, Søren W. Gersting

**Affiliations:** ^1^ Dr. von Hauner Children’s Hospital, Ludwig-Maximilians-University, Munich, Germany; ^2^ University Children’s Research, University Medical Center Hamburg-Eppendorf, Hamburg, Germany; ^3^ Institut für Physiologische Chemie, Medizinische Fakultät der Ruhr-Universität Bochum, Bochum, Germany; ^4^ Laboratory Genetic Metabolic Diseases, Academic Medical Center, University of Amsterdam, Amsterdam, Netherlands; ^5^ Children’s Hospital, University Medical Center Hamburg-Eppendorf, Hamburg, Germany

**Keywords:** network medicine, edgetic perturbations, PEX26, BRET, peroxisome

## Abstract

Peroxisomes share metabolic pathways with other organelles and peroxisomes are embedded into key cellular processes. However, the specific function of many peroxisomal proteins remains unclear and restricted knowledge of the peroxisomal protein interaction network limits a precise mapping of this network into the cellular metabolism. Inborn peroxisomal disorders are autosomal or X-linked recessive diseases that affect peroxisomal biogenesis (PBD) and/or peroxisomal metabolism. Pathogenic variants in the *PEX26* gene lead to peroxisomal disorders of the full Zellweger spectrum continuum. To investigate the phenotypic complexity of PEX26 deficiency, we performed a combined organelle protein interaction screen and network medicine approach and 1) analyzed whether PEX26 establishes interactions with other peroxisomal proteins, 2) deciphered the PEX26 interaction network, 3) determined how PEX26 is involved in further processes of peroxisomal biogenesis and metabolism, and 4) showed how variant-specific disruption of protein-protein interactions (edgetic perturbations) may contribute to phenotypic variability in PEX26 deficient patients. The discovery of 14 novel protein-protein interactions for PEX26 revealed a hub position of PEX26 inside the peroxisomal interactome. Analysis of edgetic perturbations of PEX26 variants revealed a strong correlation between the number of affected protein-protein interactions and the molecular phenotype of matrix protein import. The role of PEX26 in peroxisomal biogenesis was expanded encompassing matrix protein import, division and proliferation, and membrane assembly. Moreover, the PEX26 interaction network intersects with cellular lipid metabolism at different steps. The results of this study expand the knowledge about the function of PEX26 and refine genotype-phenotype correlations, which may contribute to our understanding of the underlying disease mechanism of PEX26 deficiency.

## Introduction

Peroxisomes are dynamic organelles formed by a single lipid membrane that encompass a matrix of mostly metabolic enzymes, and they can respond to changes in cellular homeostasis with alteration of their size, shape, and *de novo* synthesis (Schrader et al., 2016; Joshi et al., 2017). Peroxisomes take over specific tasks in lipid and reactive oxygen species (ROS) metabolism. They are involved in other metabolic and signaling pathways including antiviral response (Wanders, 2014; Ferreira et al., 2019), and they functionally and physically interact with other organelles, such as the endoplasmic reticulum and mitochondria (Schrader et al., 2013). Peroxisomal function may be impaired with aging, therefore deterioration of peroxisomal metabolism contributes to the pathogenesis of a variety of common diseases (Cipolla and Lodhi, 2017).

Inborn peroxisomal disorders are autosomal or X-linked recessive diseases either affecting peroxisomal biogenesis (PBD) or arising from single peroxisomal protein deficiencies virtually all associated with neurological impairment of varying nature and different degrees.

The group of PBD consists of the Zellweger spectrum continuum comprising, with decreasing severity, early fatal Zellweger syndrome (ZS), neonatal adrenoleukodystrophy (NALD), and infantile Refsum disease (IRD), Heimler syndrome (Ratbi et al., 2015), and the distinct clinical entity of rhizomelic chondrodysplasia punctata (RCDP) type 1. These disorders are associated with developmental brain defects as well as skeletal and craniofacial abnormalities, liver dysfunction, sensorineural hearing loss, and retinal dystrophy (Steinberg et al., 2006; Wanders and Waterham, 2006). Fourteen *PEX* genes, including peroxisome biogenesis factor 26 (*PEX26*), have been identified as the causes of PBDs (Ebberink et al., 2011; Waterham and Ebberink, 2012; Fujiki et al., 2020). The prototype of a single peroxisomal protein deficiency is X-linked adrenoleukodystrophy (X-ALD) caused by pathogenic variants in the *ABCD1* gene*.* The disorder may cause a variety of clinical symptoms ranging from adrenal insufficiency via slowly progressive paraparesis to rapidly progressive demyelination ending up in a vegetative state (Moser et al., 2007). Accumulation of very long chain fatty acids (VLCFA) and branched chain fatty acids (BCFA) in nervous tissue and impaired plasmalogen biosynthesis are hallmarks of peroxisomal dysfunction (Wanders, 2004; Wanders and Waterham, 2006). Interestingly, it has been shown that plasmalogens modulate the pathology in peroxisomal disorders with these fundamental structural phospholipids protecting cells from damage caused by VLCFA accumulation (Brites et al., 2009).

In peroxisomal disorders, genotype-phenotype correlation is often weak (Powers et al., 1992; Powers et al., 2000). This is particularly true for X-ALD, where pathogenic variants in the *ABCD1* gene may lead to multiple different clinical phenotypes including the severe cerebral forms, adrenomyeloneuropathy, or adrenal insufficiency preceding neurological disease (Moser et al., 2000). This caused the search for modifier genes or other factors influencing the clinical phenotype of X-ALD and other peroxisomal disorders (Semmler et al., 2009; Barbier et al., 2012; Galea et al., 2012).

Among the PBD, PEX26 deficiency, in particular in the presence of missense variants, displays high phenotypic variability and a weak genotype-phenotype correlation with presentation of all clinical phenotypes of the Zellweger spectrum (Fujiki et al., 2020). PEX26 is a peroxisomal membrane protein that functions as a membrane anchor for the PEX1-PEX6 complex (Matsumoto et al., 2003a). According to the Global Variome shared LOVD system, at least 23 pathogenic or likely pathogenic missense/nonsense variants in *PEX26* have been reported to date (Vihinen et al., 2012)*.* Loss-of-function variants of *PEX1*, *PEX6*, or *PEX26* were shown to completely impede matrix protein import and thus abolish all peroxisomal metabolic functions by disturbed recycling of the peroxisomal PTS1 receptor resulting in the clinical phenotype of ZS (Geisbrecht et al., 1998; Matsumoto et al., 2003b). In light of high phenotypic variability observed in the presence of missense variants, limiting PEX26 function to binding of PEX1-PEX6 and PTS1-dependent matrix protein import does not provide a sufficient explanation for the disease mechanism underlying PEX26 deficiency. This assumption is reinforced by the existence of a splice variant (PEX26Δex5) that functionally complements PEX26 deficient cells despite its localization outside the peroxisome (Weller et al., 2005). Recent studies have shown that PEX26 acts as a scaffold protein, helping to recruit the PEX13-PEX14 docking complex to peroxisomes (Tamura et al., 2014; Guder et al., 2019). All this suggests a more complex role of PEX26 in biogenesis and function of peroxisomes possibly involving interactions with proteins other than the well-characterized PEX1-PEX6 complex and the cytosolic chaperone PEX19 (Halbach et al., 2006).

The relationships of genotype and phenotype often arise from various pathobiological processes that interact in a complex network comprising gene regulation, protein interaction, and metabolite flux (Vidal et al., 2011; Sahni et al., 2015). Network medicine is a platform to explore the molecular complexity of a particular disease, leading to the identification of modules and pathways, and the molecular relationships among apparently distinct phenotypes (Barabasi et al., 2011; Mosca et al., 2015). Network-based analyses may help to get insight into genotype-phenotype relationships on the levels of molecular and biochemical parameters as well as clinical signs/features.

We performed a combined organelle protein interaction screen and network medicine approach 1) to analyze whether PEX26 establishes interactions with other peroxisomal proteins, 2) to perform fine mapping of PEX26 in the peroxisomal interaction network, 3) to answer the question whether PEX26 is involved in further processes of peroxisomal biogenesis and metabolism, 4) to characterize the interactions of PEX26, and 5) to investigate whether variant-specific disruption of protein-protein interactions (edgetic perturbations) in the global context of the peroxisomal interaction network may contribute to phenotypic variability in PBD caused by PEX26 deficiency.

Application of this approach led to the discovery of 14 novel protein-protein interactions for PEX26 and revealed a hub position of PEX26 inside the peroxisomal interactome. By analysis of edgetic perturbations of PEX26 variants, we delineated a strong correlation between the number of affected protein-protein interactions and the molecular phenotype of matrix protein import. In the functional context, the role of PEX26 in peroxisomal biogenesis was expanded encompassing matrix protein import, division and proliferation, and membrane assembly. Moreover, novel interactions of PEX26 with proteins involved in fatty acid metabolism has put PEX26 at the crossroads of VLCFA and plasmalogen metabolism, essential pathways for the development of brain pathology in peroxisomal disorders. These results expand the knowledge about the function of PEX26, a peroxisomal membrane protein involved in severe human neurological disease and refine genotype-phenotype correlation toward a better understanding of the underlying disease mechanism.

## Materials and Methods

### Plasmids

Disease causing missense variants of *PEX26* (*p*.Leu44Pro, *c*.131T > C; *p*.Leu45Pro, *c*.134T > C; *p*.Gly98Arg, *c*.265G > A; *p*.Arg98Trp, *c*.292C > T; *p*.Pro117Leu, *c*.350C > T; *p*.Pro118Arg, *c*.353C > G; *p*.Leu153Val, c.457C > G) were introduced into *PEX26* (NM_001127649.1) by site-directed mutagenesis. Truncated fragments derived from disease causing nonsense variants (*p*.Met1Thr, aa96-305; *p*.Trp99Ter, aa1-99; *p*.Arg192Ter, aa1-192) as well as artificial fragments (aa1-29; aa1-251; aa1-269; aa175-305; aa175-251; aa270-305; aa29-174) and PEX26Δex5 were amplified by conventional PCR. ORFs for the library of 90 peroxisomal proteins (Supplementary Table S2) were obtained as entry clones from a copy of the Mammalian Gene Collection or the plasmID database, or amplified by PCR. Coding sequences of *PEX26* WT, the *PEX26* variants constructs, *PEX26Δex5*, the truncated *PEX26* constructs and the all other peroxisomal ORFs were cloned by recombination (Invitrogen, Darmstadt, Germany) into BRET expression vectors coding for N- and C-terminal fusion proteins with Rluc or YFP.

### BRET Experiments

Protein-protein interactions were analyzed in living cells by bioluminescence resonance energy transfer (BRET) as described before (Gersting et al., 2012; Hillebrand et al., 2012; Lotz-Havla et al., 2021). HEK293 cells were co-transfected by electroporation (Amaxa 96-well shuttle system, Lonza) with two genes of interest either fused to *Rluc* (donor) or *YFP* (acceptor) at an acceptor to donor ratio of 3:1. After 48 h coelenterazine (30 μM, PJK) was added to the living cells and light emission was collected in a 96-well microplate luminometer (LUMIstar OPTIMA, BMG Labtech) for 10 s at 475 nm (Rluc signal) and 535 nm (BRET signal). The BRET ratio was calculated based on *R* = *I*_*A*_ */I*_*D*_ – *cf*, where *R* is the BRET ratio, *I*_*A*_ is the intensity of light emission at 535 nm, *I*_*D*_ is the intensity of light emission at 475 nm, and *cf* is a correction factor (BRET_control_/Rluc_control_) with the negative control of donor fusion-proteins co-expressed with YFP in the absence of the second protein of interest. For each protein pair, all eight possible combinations of N- or C-terminal fusion proteins of the two proteins of interest were investigated. Each combination was tested in duplicates. An interaction of a protein pair investigated was assumed if at least one out of eight tested combinations resulted in a BRET ratio above the method-specific threshold of 0.094. All interactions found were confirmed in at least two additional independent experiments. A positive control interaction (bJun-bFos) and the expression of YFP-Rluc as a positive control construct were included in every individual experiment.

BRET experiments were performed for 1) PEX26 wild-type and variants with PEX6, 2) PEX26 wild-type with peroxisomal proteins of the organelle library, 3) PEX26Δex5 and truncated PEX26 fragments with PEX26 interaction partners, and 4) variant PEX26 with PEX26 interaction partners.

### Real-Time PCR

Real-time PCR was performed to analyze the relative mRNA expression of *FIS1 in PEX26* deficient fibroblast of a patient with the severe phenotype of Zellweger syndrome (GM17398 (Weller et al., 2005)) compared to healthy primary cultured fibroblasts. Fibroblasts were cultured in Dulbecco’s modified Eagle medium high-glucose supplemented with 10% fetal bovine serum at 37°C under 5% CO2 and 95% air. Total RNA was isolated from fibroblasts with TRI^®^ Reagent from Applied Biosystems/Ambion followed by phenol–chloroform extraction according to the manufacturer’s instructions. For cDNA synthesis 1 μg of total RNA and the High Capacity cDNA Reverse Transcription Kit (Life Technologies/Applied Biosystems) were used. For real-time PCR, TaqMan™ Gene Expression Assays (Life Technologies/Applied Biosystems) including predesigned probes and primer sets for human *FIS1* (Hs00211420_m1) and human *ACTB* (Hs99999903_m1) were used. PCR reactions using Maxima™ Probe qPCR Master Mix (Thermo Scientific/Fermentas) were analyzed with the Mx3000P (Stratagene). The relative expression of *FIS1* mRNA was analyzed in *n* = 3 different isolations, and normalized to the level of *ACTB* mRNA in the same cDNA using the comparative CT method (2^–ΔΔCT^) (Livak and Schmittgen, 2001).

### Computational Analysis

Integrated disease network was depicted by a spring-embedded layout of the Cytoscape 2.8.2. software (Kamada and Kawai, 1989). Protein-protein interaction network topology was modeled using the edge-weighted force-directed layout of the Cytoscape 2.8.2. software (Smoot et al., 2011). Interactions identified in this study were merged with a dataset of known peroxisomal PPI (*n* = 67). Information on known peroxisomal interactions was extracted from leading PPI databases (BOND, Biogrid, String, HPRD, MIPS) and subsequently manually curated to eliminate faulty entries and to account for PPI listed in the PubMed database that were not represented in the databases. Betweenness centrality as a measure of the number of shortest paths passing the node was visualized with low values to small size. The Cytoscape network analyzer plugin was used to determine network parameters (clustering coefficient, number of connected components, network centrality, average number of neighbors, number of nodes, network density, network heterogeneity, number of isolated nodes) of the induced peroxisomal subgraph with all nodes selected that are directly or indirectly associated with the PEX26 network (Supplementary Table S4). For hierarchical cluster analysis, the distance matrix algorithm of the *R* open source software package (hClust) was applied. Heatmap analysis was performed by the Microsoft Excel software with an implemented Excel-add-in macro.

### Biochemical Score Generation

To analyze phenotypic parameters as a function of the number of maintained interactions for WT and variant PEX26, a biochemical score was calculated based on data from literature (Matsumoto et al., 2003b; Furuki et al., 2006) reflecting the import of peroxisomal matrix proteins and protein stability, respectively. For this, protein amount (Furuki et al., 2006), catalase import, PTS1-dependent import, and PTS2 dependent import (Matsumoto et al., 2003b) were given a score of 30–10–10–10–10, respectively, yielding a total of 60 for WT PEX26. For the protein amount, a value of 30 reflects a protein stability comparable to the WT PEX26 and a score of 15 reflects reduced but residual protein stability. For peroxisomal matrix protein import a score of 10 corresponds to 100% of matrix protein import (see also Supplementary Table S6).

### Statistical Analyses

Statistical analyses were performed using the GraphPad Prism 5 Software. A Pearson correlation analysis was performed to analyze correlations between the number of protein-protein interactions for WT and variant PEX26 derived from BRET experiments, and the respective biochemical score. Curve fitting using linear regression analysis was applied to assess the relationship of variables in correlation analysis.

To compare relative mRNA levels of *FIS1* in PEX26 deficient fibroblasts (GM17398) and healthy control fibroblasts an unpaired *t*-test was used.

## Results

### Interaction of Mutant PEX26 With PEX6 and Genotype-Phenotype Correlations

**FIGURE 1:**
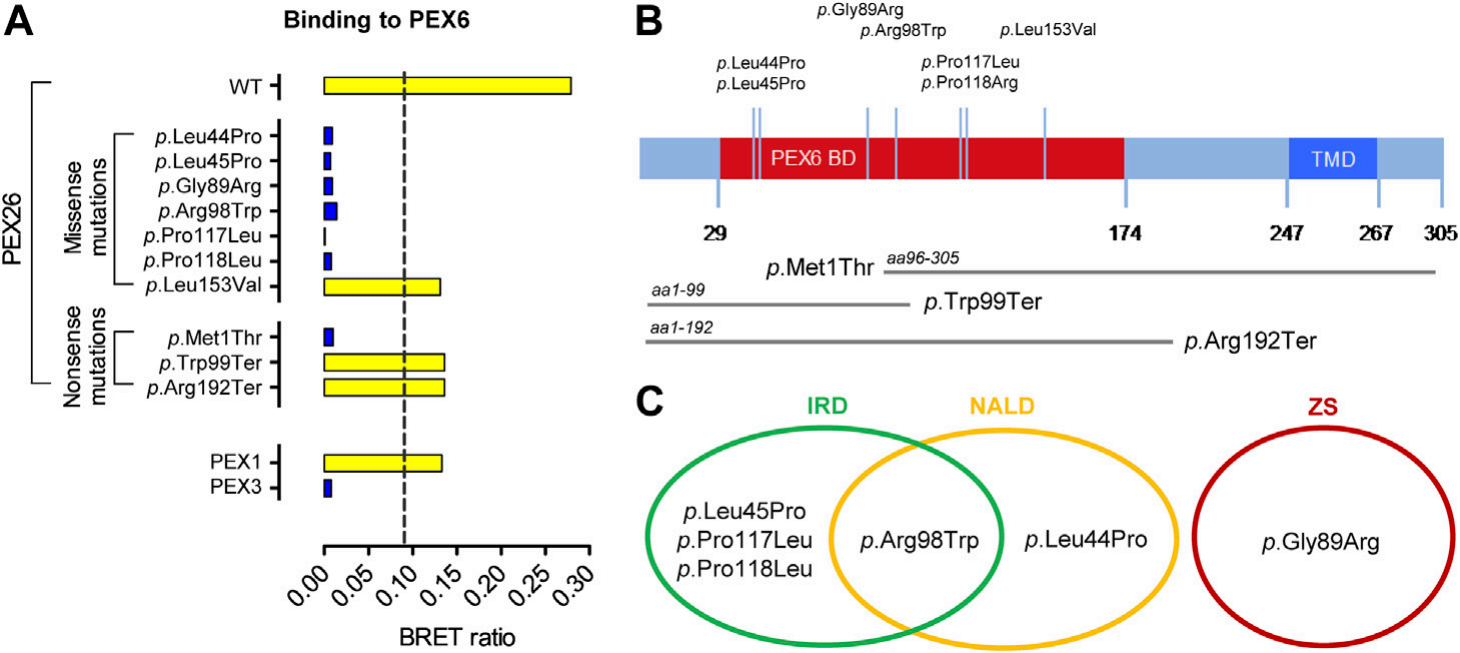
Pathogenic variants in *PEX26* and their impact on the interaction with PEX6. **(A)** Impact of side-chain replacements (*p.*Leu44Pro, *p.*Leu45Pro, *p.*Gly89Arg, *p.*Arg98Trp, *p.*Pro117Leu, *p.*Pro118Arg, *p.*Leu153Val) and truncated fragments derived from nonsense variants (*p*.Met1Thr, aa96–305; *p*.Trp99Ter, aa1–99; *p*.Arg192Ter, aa1–192) on PEX26 binding to PEX6 analyzed by BRET (positive interaction, yellow bar; non-interacting, blue bar). The dashed line highlights a method specific threshold for positive PPI of 0.094. A positive interaction of PEX6 with PEX1 and no interaction of PEX6 with a putatively non-interacting protein (PEX3) are shown as a control. **(B)** Sequence annotations of PEX26 demonstrating the PEX6 binding domain (BD), the transmembrane domain (TMD), the topology of pathogenic missense variants investigated in this study, and the truncated fragments derived from disease causing nonsense variants. **(C)** Association of the pathogenic missense variants with phenotypes of the Zellweger spectrum: Zellweger syndrome (ZS), neonatal adrenoleukodystrophy (NALD), and infantile Refsum disease (IRD).

Physical interaction with the peroxin PEX6 is a key function of the PEX26 protein (Matsumoto et al., 2003a). To determine a potential variant induced impairment on this process, interactions of wild-type and variant PEX26 with PEX6 were analyzed by means of bioluminescence resonance energy transfer (BRET). The application of BRET allows for interaction analyses in living cells in the physiological environment for the proteins investigated (Gersting et al., 2012; Lotz-Havla et al., 2021). We investigated seven missense variants in *PEX26* (Figure 1A), which all mapped to the PEX6 binding domain of PEX26 (Weller et al., 2005; Nashiro et al., 2011; Fujiki et al., 2020) and three truncating or nonsense variants (Figure 1B). Most missense variants showed a disrupted interaction with PEX6. For the PEX26 variant (*p*.Leu153Val) an interaction with PEX6 was determined, however, at a lower BRET ratio when compared to the wild-type. In addition, we analyzed known patient variants in *PEX26* that result in truncated proteins. The M1T variant (aa96–305) shows N-terminal truncation, which affects the N-terminal part of the PEX6 binding domain. The variant W99X (aa1–99) displays a partially restored and the R192X variant (aa1–192) a fully restored PEX6 binding domain. For the M1T variant, no interaction with PEX6 was observed while an interaction was detected for W99X and R192X (Figure 1A).

### BRET-Based PPI-Screen of PEX26 Against a Peroxisomal Library

**FIGURE 2:**
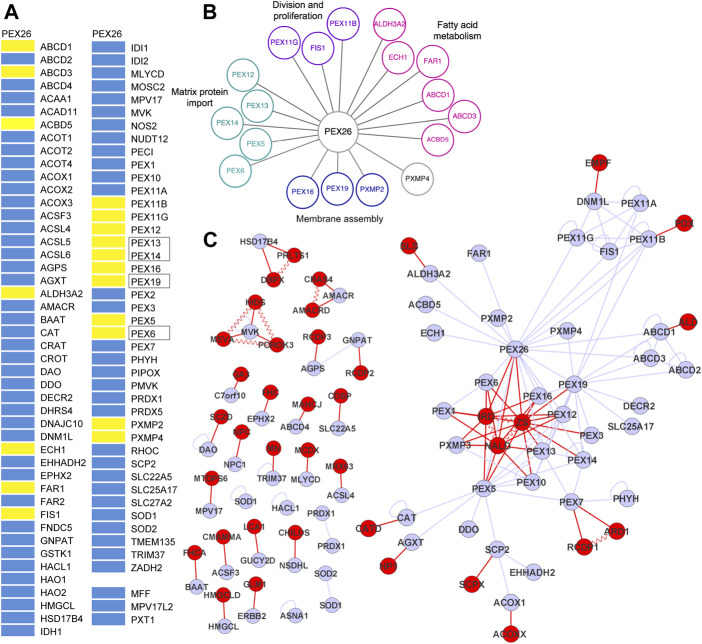
PEX26 interactions with the peroxisomal interactome. **(A)** BRET-based PPI screen of PEX26 WT against a peroxisomal protein library (positive interaction, yellow bar; non-interacting, blue bar; known interaction, black frames). **(B)** Protein interactions with PEX26 WT are grouped with respect to peroxisomal function. **(C)** Integrated disease network demonstrating the relation of peroxisomal proteins (grey nodes, peroxisomal proteins; grey edges, protein-protein interactions) and peroxisome associated disorders (red nodes, disorders; red edges, phenotype gene relationships; vermiculated red line: disease-disease interactions).

Next, we searched for potential other interaction partners of PEX26. A bioluminescence resonance energy transfer based screen of PEX26 against an organelle library of the peroxisome (*n* = 90) was performed (Supplementary Table S2). The peroxisomal library consists of 87 proteins with peroxisomal annotation of the peroxisome database (Schluter et al., 2010) and three additional proteins (MFF, MPV17L2, PXT1) that are discussed to display peroxisomal location (Palmer et al., 2013; personal communication; Kaczmarek et al., 2011). The peroxisomal library covered 88% of all proteins annotated with peroxisomal localization (Schluter et al., 2010). We identified 14 novel interactions of PEX26 with 13 peroxisomal membrane proteins and 1 peroxisomal matrix protein, respectively, while known interactions with PEX6, PEX19, PEX14 and PEX13 were confirmed (Figure 2A). To analyze PEX26 function in the context of global peroxisomal biogenesis and metabolism, we determined functional annotations of the PEX26 interaction partners with respect to the gene ontology term <biological process> (Ashburner et al., 2000). First order interaction partners of PEX26 are involved in distinct non-overlapping processes (matrix protein import, division and proliferation, fatty acid metabolism and membrane assembly) where PEX26 establishes 13 interactions with proteins not associated with peroxisomal matrix protein import (Figure 2B). Thus, in addition to the latter function, the PEX26 is linked to processes of peroxisomal *de novo* synthesis, proliferation, and metabolic function.

### Modeling of Peroxisomal Network Topology

We analyzed the organizing principles of the network of interactions of peroxisomal proteins in order to get insight into functional relations within this network (Barabasi et al., 2011).

First, all interactions identified in this study were merged with a dataset of known peroxisomal protein interactions (*n* = 67) and the peroxisomal network topology was modeled. Among all peroxisomal proteins (nodes) and interactions (edges), an induced PEX26 sub-network comprises all nodes (*n* = 37) that are directly or indirectly linked to PEX26 by edges (*n* = 74) and covers 40% of all nodes, and 90% of all edges within the peroxisomal interactome. Graph-theoretic modeling revealed that PEX26 1) shows highest number of individual edges, 2) is a hub protein, and 3) is involved in three network motifs. The PEX26 protein takes a central position within this network and establishes 18 binary edges. This has two major implications for function and dysfunction of PEX26. First, PEX26 occupies a bottle-neck position within the peroxisomal interactome and displays the highest value of betweenness centrality (0.46) followed by PEX5 (0.41), PEX19 (0.20), and PEX7 (0.19). This parameter is a measure of shortest paths inside a network and correlates with essentiality and disease relevance of a protein (Goh et al., 2007). Second, we observed cross-linking of three network motifs by PEX26. Network motifs are groups of nodes representing highly interconnected subgraphs inside a network that have been shown to contain functional building blocks of proteins of similar pathways or cellular functions (Milo et al., 2002; Przulj, 2011). In detail, three areas of highly interconnected nodes were observed comprising the ABCD1-3 proteins, PEX11A/B/G with FIS1 and DNM1L, and PEX5/7/10/12/13/14, respectively.

### Results From Disease Network Analysis

To analyze the relation of peroxisomal proteins and peroxisome associated disorders, an integrated protein-protein-interaction and disease network was constructed. We analyzed this network with respect to 1) topological location of peroxisomal proteins and disorders, 2) local clustering of disease genes forming modules, and 3) the role of PEX26 and interactions partners within this network.

An OMIM database research was performed using the search terms <peroxisom*> and the 87 proteins from our peroxisomal library (Supplementary Table S2). We obtained a list of peroxisome related disorders, disease genes, and associations between them and identified 65 phenotype gene relationships with known molecular basis (Supplementary Table S3). These were associated with 40 different clinical phenotypes, with few exceptions neurological disorders. The different phenotype gene relationships were analyzed with respect to shared genes in order to define disease-disease interactions (Supplementary Table S3). The integrated interaction and disease network based on the interactome of peroxisomal protein-protein interactions, the phenotype gene relationships, and the disease-disease interactions contained 104 nodes establishing 158 edges (Figure 2C). Application of the Kamada-Kawai algorithm generated a spring-embedded layout (Kamada and Kawai, 1989) with a main network composed of 51 nodes and 119 edges and 25 additional connected components or sub-networks not linked to the main network. Nodes representing the phenotypes of the Zellweger spectrum (IRD, NALD, ZS) took a central position inside the main network whereas all other disease nodes were positioned at the margins. In order to gain a better understanding of how peroxisomal diseases are related to each other as well as to peroxisomal proteins, we performed a cluster analysis of the main integrated network using MCL cluster (Enright et al., 2002; Koh et al., 2012). The resulting degree of clustering (clustering coefficient 0.35) implied the existence of topological modules of highly interlinked local regions. These modules each represent a group of network components that together contribute to a cellular function and its disruption results in a particular disease phenotype. Following the “local hypothesis”, proteins involved in the same disease have an increased tendency to interact with each other while the corollary of this hypothesis implies that pathogenic variants in interacting proteins often lead to similar disease phenotypes (Barabasi et al., 2011). We identified five modules with the largest network centered on PEX26. This network area contained the complete motif comprising proteins involved in division and proliferation (PEX11A/B/G, FIS1, and DNM1L) and in part the matrix protein import motif (PEX5/6/7/10/12/13/14) as well as nodes representing both Zellweger spectrum diseases and peroxisomal division defects.

### Functional Significance of PEX26 Interactions

**FIGURE 3:**
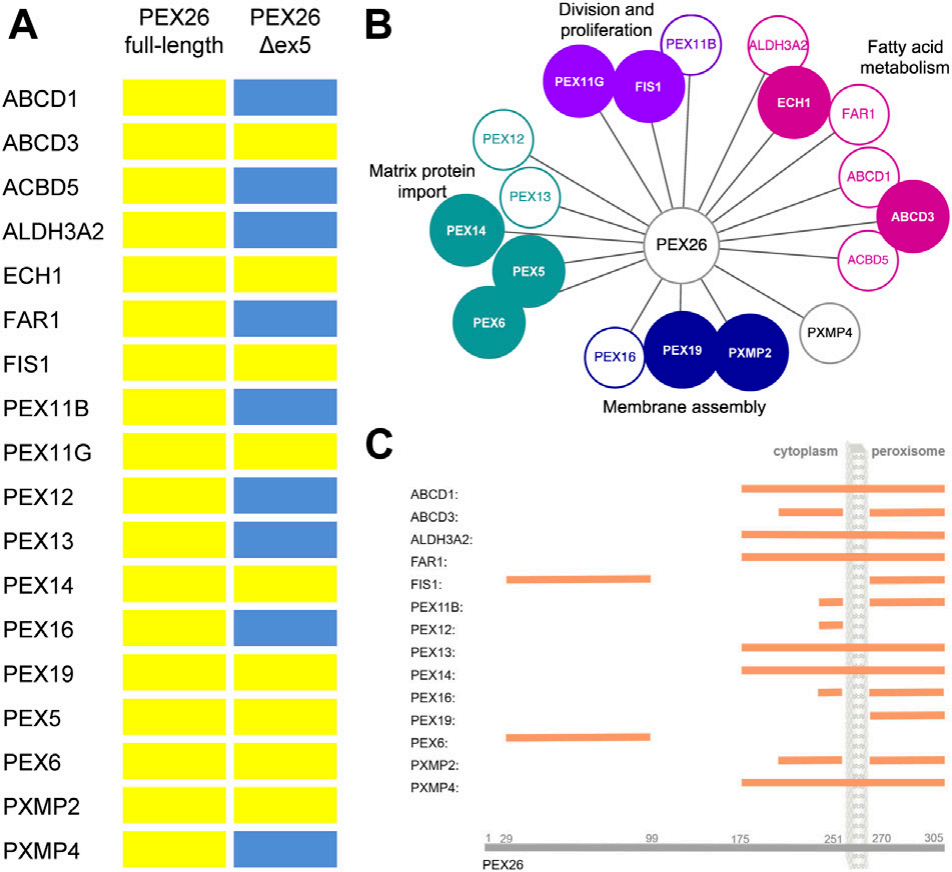
Characterization of PEX26 interactions. **(A)** BRET-based PPI screen of the splice variant PEX26Δex5 against the known and newly identified interaction partners of PEX26 full-length (positive interaction, yellow bar; negative interaction, blue bar). **(B)** Protein interactions with PEX26 are again grouped with respect to peroxisomal function. Proteins that interact with both, the full-length PEX26 protein and the splice variant PEX26Δex5 are highlighted by filled nodes, proteins interacting with the full-length PEX26 only are depicted with empty nodes. **(C)** Binding domains of PEX26 to its interaction partners determined by BRET analysis are demonstrated (orange bar) with respect to the topology. No binding domain was determined for ACDB5, ECH1, PEX11G, and PEX5 as repetitive testing resulted in conflicting assertions.

The splice variant PEX26Δex5 has been described to complement PEX26-deficient cells as efficiently as does the full-length protein (Weller et al., 2005). To further evaluate the functional significance of the newly identified interactions, a BRET-based PPI screen was performed probing the interaction of PEX26Δex5 against the interaction partners of PEX26 (Figure 3A). As observed for the full-length protein, the splice variant showed interactions with candidate proteins of all four peroxisomal processes (matrix protein import, division and proliferation, fatty acid metabolism and membrane assembly) (Figure 3B). However, PEX26Δex5 interacted only with ABCD3, ECH1, FIS1, PEX11G, PEX14, PEX19, PEX5, PEX6, and PXMP2. Hence, not all interactions of the full-length protein could be confirmed for the splice variant. The PEX26Δex5 splice variant is lacking aa 223–271, therefore, we aimed to investigate potential binding domains of PEX26 that establish interactions with proteins identified in the screen. We performed BRET experiments using artificial truncated fragments of PEX26 (aa1–19; aa1–251; aa1––269; aa29–174; aa175–251; aa175–305; aa270–305) and truncated fragments derived from disease causing nonsense variants *p*.Trp99Ter (aa1–99), *p*.Arg192Ter (aa1–192), and *p*.Met1Thr (aa96–305) (Figure 3C; Supplementary Figure S1). The known N-terminal binding domain of PEX26 to PEX6 (Weller et al., 2005; Nashiro et al., 2011) and its C-terminal binding domain to PEX19 (Halbach et al., 2006) were confirmed. Interestingly, except for interaction with PEX6 and FIS1, binding domains covering all other interaction partners mapped to the area of aa 175–305.

### *PEX26* Variant-Induced Edgetic Perturbations of the Peroxisomal Interactome

**FIGURE 4:**
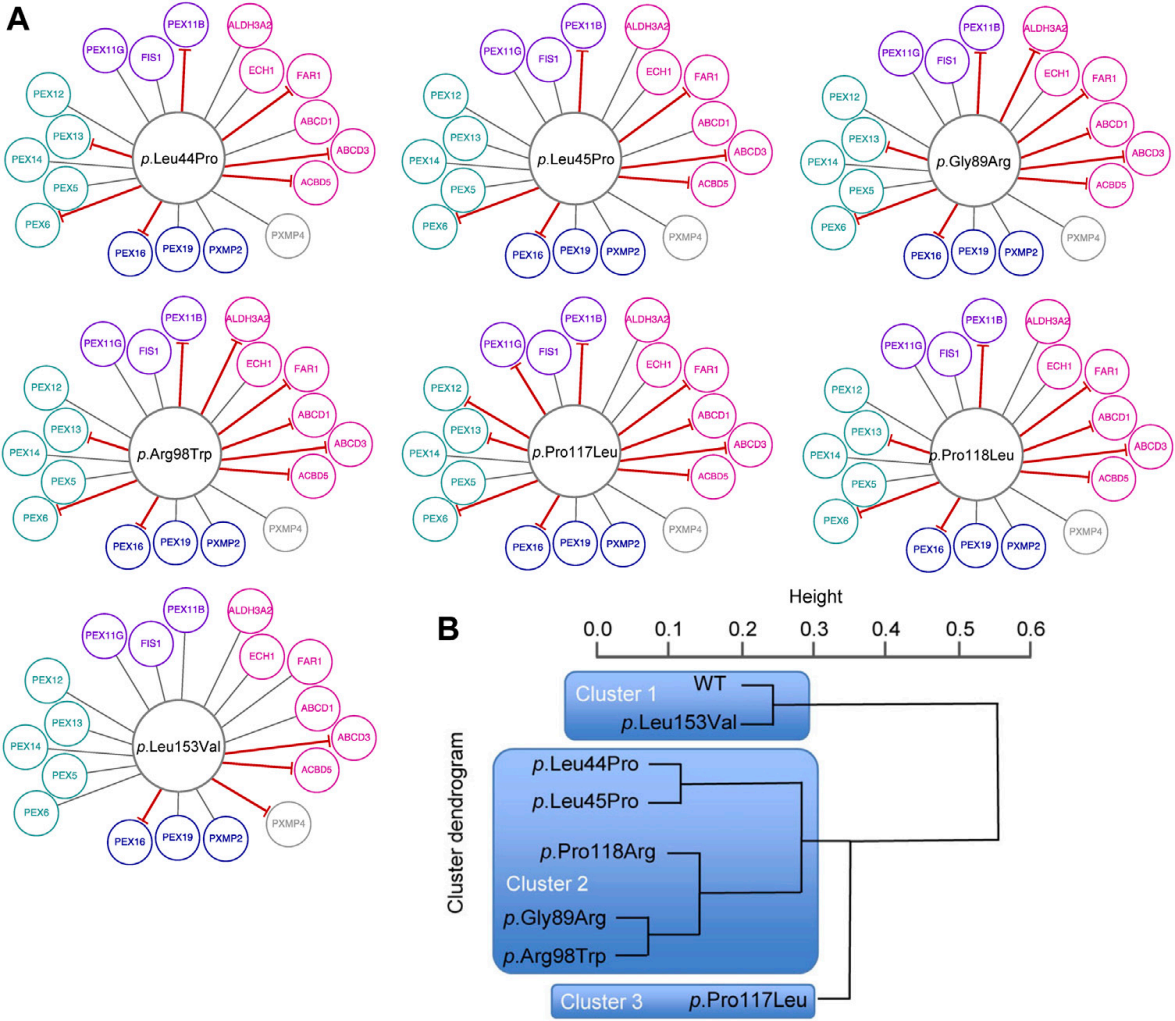
Edgetic perturbations due to missense variants in *PEX26*. **(A)** Variant specific PEX26 networks vary with respect to number and pattern of edgetic perturbations. Interacting proteins are grouped with respect to peroxisomal function (fatty acid metabolism, pink; membrane assembly, dark blue; matrix protein import, cyan; division and proliferation, violet; unknown function, grey). Disrupted interactions are depicted by a red T-arrowed edge. **(B)** Hierarchical cluster analysis defined subgroups of missense variants using a distance matrix algorithm. The height of the dendrogram reflects the relation of individual clusters. Three main clusters are highlighted by blue boxes.

For a fine mapping of the effect of individual missense variants on the peroxisomal network, interactions between variant PEX26 and PEX26 binding proteins were determined by BRET analyses. We observed that different missense variants in the *PEX26* gene lead to a varying reduction in the number of edges, ranging from four losses (*p*.Leu153Val) to ten losses (*p*.Pro117Leu) (Figure 4A). In addition, we observed different patterns of edge-loss distributions with respect to the network motifs (Figure 4A). To classify variants based on the number and the pattern of edgetic perturbations, we performed a hierarchical cluster analysis. A protein interaction matrix (Supplementary Table S4) revealed that all variants showed positive interactions with the proteins PEX5/14/19, ECH1, FIS, and PXMP2, whereas interactions with PEX16, ABCD3, and ACBD5 were perturbed. The nine remaining edges are linked to the processes of fatty acid metabolism, matrix protein import, and division and proliferation but not to membrane assembly. This defined three main clusters with three subgroups within the second cluster (Figure 4B). Cluster 1 represented wild-type PEX26 and the variant *p*.Leu153Val that did not show any perturbed interaction to the building-block of matrix protein import. However, the *p*.Leu153Val variant induced a loss of interaction with PXMP4, a protein of unknown function. This is in line with the biochemical phenotype for this variant where matrix protein import was not significantly affected (Supplementary Table S1). The *p*.Pro117Leu represented cluster 3 with the most pronounced edgetic perturbations. For this variant, besides those interactions that are conserved for all variants, only interactions with ALDH3A2 and PXMP4 were detected. Thus, all four building-blocks were affected with the most significant impact on matrix protein import as compared to the other variants. These findings are reflected by a severe biochemical phenotype for this variant that displays no residual matrix protein import (Supplementary Table S1). Global discrimination criteria of cluster 2 from cluster 1 are the perturbation of interactions with PEX6, as well as with PEX11B and FAR1. Hence, edgetic perturbations of all network motifs were observed, but to a lesser extent than for cluster 3. Further differentiation within cluster 2defines the subgroup *p*.Leu44Pro/*p*.Leu45Pro that maintains interactions to ABCD1 and ALDH3A2, the intermediate subgroup *p*.Pro118Arg that maintains only the interaction to ALDH3A2, and the subgroup *p*.Gly89Arg/*p*.Arg98Trp shows loss of both interactions. Thus, the subgroups inside cluster 2differ with respect to the extent of edgetic perturbations to the building-block of fatty acid metabolism.

**FIGURE 5:**
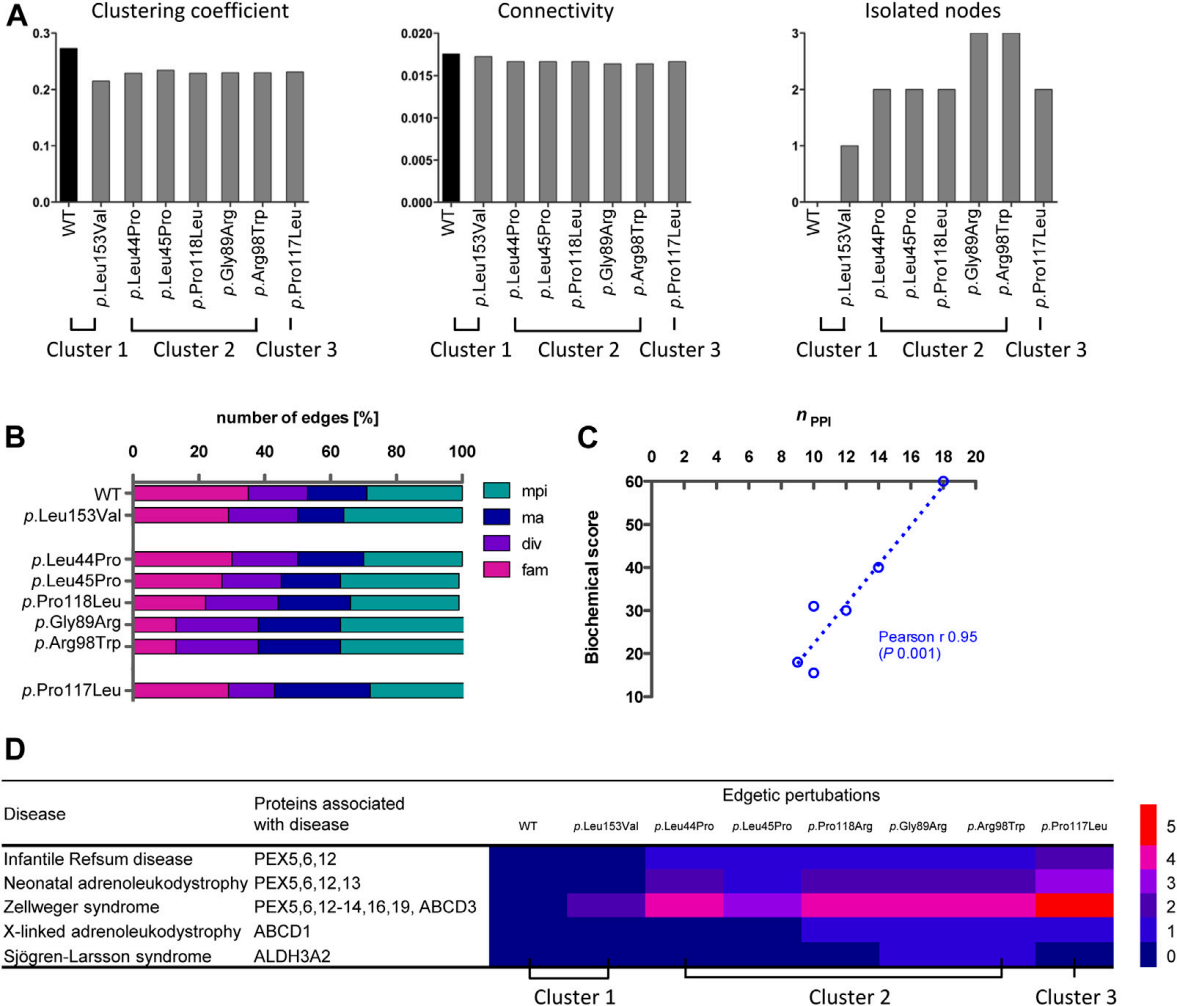
Analyses of variant-specific PEX26 networks and correlation to phenotypic parameters. **(A)** Clustering coefficient, connectivity and isolated note analysis of the WT and variant PEX26 network comprising all nodes directly or indirectly connected to WT or variant PEX26, respectively, by edges. **(B)** Impact of edgetic perturbations due to missense variants in PEX26 on building-blocks of peroxisomal function (mpi, matrix protein import; ma, membrane assembly; div, division and proliferation; fam, fatty acid metabolism). The total number of remaining edges for each variant was set to 100%. Variants are grouped according to previously identified clusters. **(C)** Correlation analysis between the number of maintained interactions (*n*_PPI_) and a literature based biochemical score reflecting the import of peroxisomal matrix proteins (catalase, PTS1, PTS2) and protein stability. The relationship of the correlating variables was linear, as depicted by the dashed line. **(D)** Association of edgetic perturbations and clinical disease entities of the respective PEX26 binding partners. The heatmap highlights the number of edgetic perturbations of the PEX26 variants to proteins associated with different disease related peroxisomal dysfunctions.

More detailed network analyses were performed in order to describe global and local properties of variant-specific PEX26 networks. With respect to global properties, no significant changes were found for the network parameter <clustering coefficient>, which is a measure of the degree to which nodes in a graph tend to cluster. Similarly, no significant changes were observed for the network parameter <connectivity>, which represents robustness of the whole network (Figure 5A; Supplementary Table S5). By contrast, the clusters differed as to the local occurrence of isolated nodes. Besides the negative interaction of *p*.Leu153Val with PXMP4, all isolated nodes mapped to the fatty acid metabolism motif. Detailed investigation of the impact of individual variants on different building-blocks confirmed impaired matrix protein import, but again fatty acid metabolism was affected to the highest extent (Figure 5B). To quantify the variant-specific impact on local network properties, the number of remaining edges was related to the biochemical phenotype. A score combining all data available on biochemical phenotypes including residual PEX26 protein amount, catalase import, and PTS1/2 dependent import (Supplementary Table S6) showed robust correlation with edgetic perturbations (Pearson *r* 0.95, *p* 0.001) (Figure 5C). To expand the genotype-phenotype correlation to clinical disease entities, a heat-map was constructed that quantified the impact of edgetic perturbations on phenotype-gene relationships (Figure 5D).

### Functional Consequences of Variants in the *PEX26* Gene

**FIGURE 6:**
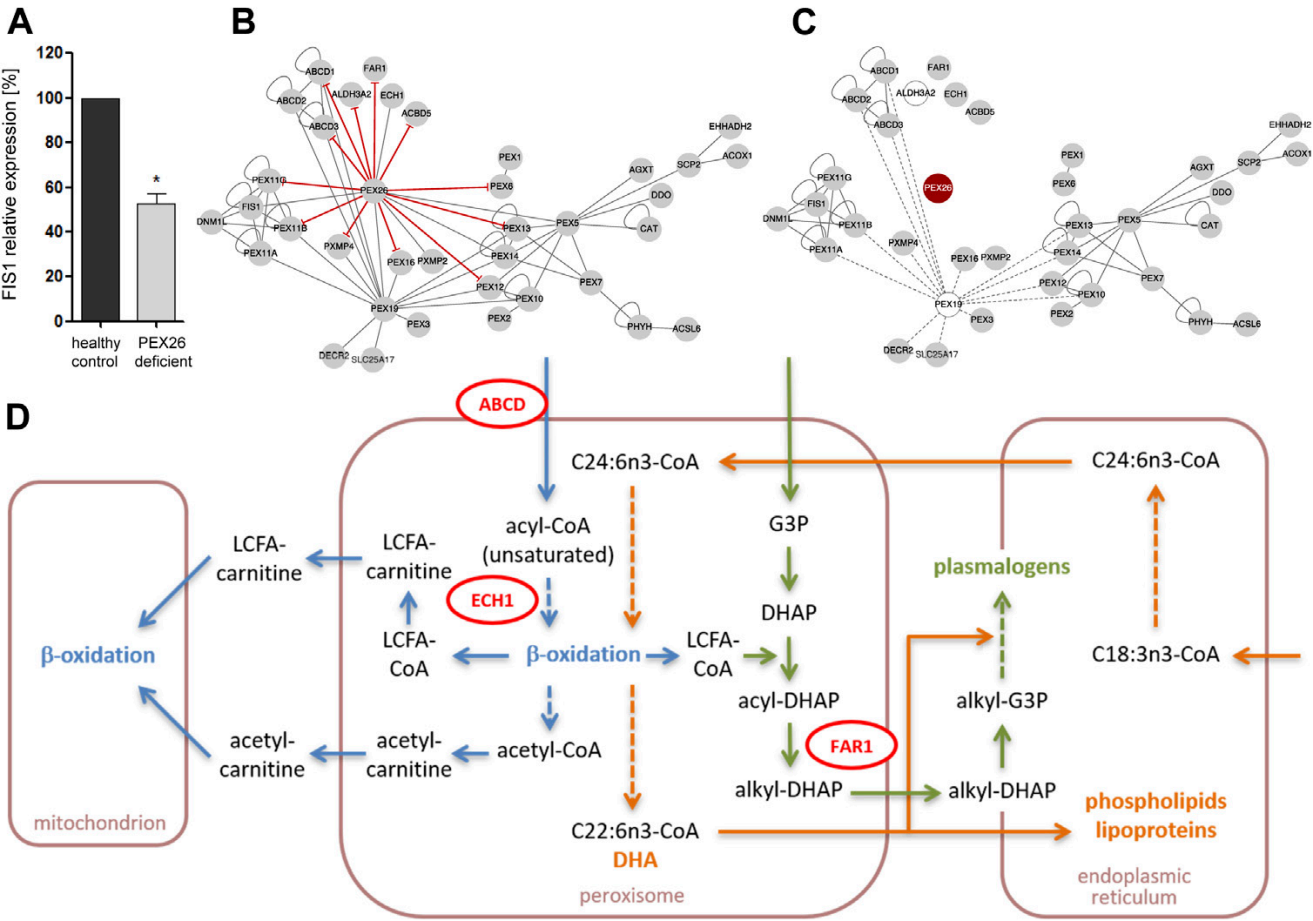
Impact of edgetic perturbations and node removal on the PEX26 network and metabolic pathways. **(A)** Relative mRNA levels of *FIS1* in PEX26 deficient fibroblasts (GM17398) and healthy control fibroblasts (*n* = 3). The expression ratios of healthy control fibroblasts were set as 100%, *p < 0.05. **(B)** The sites of missense variant induced edgetic perturbations on the PEX26 network are depicted by red T-arrowed edges. **(C)** A loss of PEX26 protein (node removal, red) disrupts the integrity of a functionally weighted PEX26 network. Proteins without contribution to intrinsic function of mature peroxisomes are depicted by clear nodes and their respective edges by dashed lines. **(D)** Schematic representation on key steps in β-oxidation (blue), docosahexaenoic acid (DHA) metabolism (orange) and plasmalogen synthesis (green) that are shared by mitochondria, peroxisomes and the endoplasmic reticulum. Direct interaction partners of PEX26 are depicted in red (ABCD1 and ABCD3 summarized as ABCD, ECH1, FAR1). The associated pathways may be affected due to secondary dysfunction of these proteins as mediated by node removal or edgetic perturbations of PEX26. VLCFA, very long-chain fatty acids; LCFA, long-chain fatty acids; CoA, coenzyme A; G3P, glycerol-3-phosphate; DHAP, dihydroxyacetone phosphate; C18:3n3/C24:6n3, precursors of DHA (C22:6n3).

To analyze whether PEX26 function plays a role for peroxisomal division and proliferation, we analyzed the impact of *PEX26* deficiency on the mRNA expression of *FIS1*. Growth and division of the peroxisomal compartment follow morphologically well-defined steps of membrane deformation/elongation, constriction and final fission (Schrader et al., 2012). FIS1 is a key component of the peroxisomal fission machinery (Koch et al., 2005) and FIS1 recruits DNM1L, the final membrane scission mediator. The expression level of FIS1 mRNA analyzed by qPCR was significant reduced (*p* = 0.015) in *PEX26* deficient fibroblast of a patient with the severe phenotype of Zellweger syndrome (GM17398) (Weller et al., 2005) as compared to a healthy fibroblast cell line (Figure 6A). Considering that peroxisomal division and proliferation is assigned to be regulated by a change in the expression of *FIS1* and *DNM1L*, we suggest a functional role of PEX26 for peroxisomal division and proliferation, an issue necessitating further investigation.

Next, we aimed to analyze the consequences of variants in the *PEX26* gene on the complete network and its functions (Figures 6B,C). The connectivity of the induced PEX26 subgraph of all peroxisomal interactions was predominantly based on multiple edges and hub positions of cytosolic PEX19 as well as of PEX26. We modeled complete disruption of all edges established by PEX26 (node removal) and observed that integrity of the remaining network relied on PEX19 interactions (Figure 6C). However, PEX19 serves as a chaperone for targeting proteins to the peroxisomal membrane and is thought not to be involved in post-biogenesis functional peroxisomal processes *per se*. Thus, a node removal of PEX26 in a functionally weighted network without PEX19 resulted in a complete breakdown of the peroxisomal interactome.

## Discussion

The technical advance and availability of genetic tools for discovery of Mendelian disease genes has significantly expanded the number of phenotype gene relationships known to date (McKusick, 2007). However, it is still a challenging task to model and understand the impact of human genetic variations on diseases (Vidal et al., 2011). Most of our current knowledge on function and dysfunction of single genes and their gene products is derived from elaborate knock-out experiments in model organisms. More than half of the disease causing variants are missense, nonsense or small insertions or deletions leading to the production of a variant protein rather than to complete protein disruption (Stenson et al., 2003). Many proteins exert their function as part of higher order complexes. Perturbation of interactions with other proteins or metabolites may have significant impact on cellular processes organized as complex molecular networks. Emerging tools of network medicine offer the opportunity to investigate the impact of individual variants on these networks. The known function of PEX26 is to bind PEX6 and by this serves as “helping hand” in a late step of peroxisomal matrix protein import. Our work was based on the assumption that impairment of the interaction of PEX26 with PEX6 is not sufficient to explain the variable clinical phenotypes associated with deficiency of this protein.

According to current knowledge, disruption of the PEX26-PEX6 interaction would lead to a loss-of-function phenotype in terms of peroxisomal matrix protein import resulting in ZS. However, missense variants in PEX26 are predominantly associated with mild clinical phenotypes of the Zellweger spectrum (Figure 1C; Supplementary Table S1). Patients harboring the *p*.Leu44Pro and *p*.Arg98Trp variant in the homozygote or compound heterozygote state displayed the mild phenotype of NALD and patients harboring the *p*.Leu45Pro, *p*.Arg98Trp, or *p*.Pro117Leu variant in compound heterozygosity with a putative null allele even presented the mildest phenotype of IRD. Only the *p*.Gly89Arg/*p*.Gly89Arg genotype was associated with the severe phenotype of ZS. In a previous study by Furuki et al. (2006) residual binding of PEX26 variants to PEX6 was found based on co-immunoprecipitation (Supplementary Table S1). However, there was no correlation between residual PEX6-binding or the biochemical phenotype of matrix protein import, respectively, and severity of the clinical phenotype of patients harboring *PEX26* missense variants (Matsumoto et al., 2003b; Steinberg et al., 2004; Weller et al., 2005; Furuki et al., 2006). A patient homozygote for *p*.Gly89Arg displayed the severe ZS clinical phenotype although expression of the *p*.Gly89Arg PEX26 variant in PEX26 deficient cells resulted in 70–90% residual PEX6 binding whereas the *p*.Pro117Leu variant in functional hemizygosity with a putative null allele (*p*.Leu153Val + *p*.Arg288fs366X) was associated with the mildest phenotype of IRD despite lower residual binding of PEX6 (40–70%). Both variants, however, induced a complete block in peroxisomal matrix protein import.

In conclusion, the apparent lack of correlation between the molecular phenotypes of PEX6-binding and matrix protein import with clinical phenotypes of PEX26 deficiency once again supports the view that function and dysfunction of PEX26 is not limited to binding of PEX6.

An organelle screen identified 14 novel interaction partners of PEX26 building a link to other peroxisomal processes. Besides the full-length protein, the splice variant PEX26Δex5 also interacts with candidate proteins of respective peroxisomal processes. However, PEX26Δex5 only interacted with nine out of 18 PEX26 interaction partners. Considering that the splice variant has been shown to complement PEX26-deficient cells as efficiently as does the full-length protein in terms of matrix protein import (Weller et al., 2005; Guder et al., 2019), we assumed that particularly the interactions of PEX26 to PEX12 and PEX13, both involved in matrix protein import, are at least not essential for its function.

Construction of a network of all known peroxisomal protein interactions merged with interactions identified in this study provided experimental and bioinformatic evidence for a more complex role of PEX26 in global peroxisome function as well as in pathogenesis of peroxisomal disorders. Modeling of the network topology revealed that PEX26 takes a central position inside the network and physically links processes of matrix protein import, division and proliferation, and lipid metabolism found to be organized as network motifs. Proteins inside each of these motifs belong to a specific biological process such as fatty acid metabolism, peroxisomal division and proliferation, or peroxisomal matrix protein import. We demonstrated that PEX26 bears network based characteristics of a disease protein, links different functions, and thus is a candidate to induce varying phenotypes by variant-specific impairment of protein interactions. Based on the assumption that network motifs constitute units of specific function, PEX26 interconnects these processes being a hub rather than sharing function with its interacting partners. The association of PEX26 with several different functional processes gave rise to the hypothesis that variants-specific impairment of these processes may contribute to the phenotypic variability of PEX26 deficiency.

The analysis of the PEX26 network in the context of peroxisomal phenotype gene relationships and disease-disease interactions enabled us to establish a link to other clinical phenotypes. Modeling of network modules by cluster analysis, associated X-ALD with the ABCD1-3 and PEX19 network motif, whereas PEX26 was grouped with all phenotypes of the Zellweger spectrum and phenotypes of deficient peroxisomal fission (PEX11B and DNM1L deficiency). mRNA expression analysis revealed an influence of PEX26 on the fission factor *FIS1* level, suggesting that PEX26 is involved in regulation of peroxisomal division and proliferation mechanisms. This is especially of interest, as the so far described peroxisomal fusion/fission complex is proposed to be part of a “signaling system” to sense the state and/or distribution of the peroxisomal populations within the cell (Bonekamp et al., 2012). Hence, one could hypothesize, that PEX26 plays a functional role in this signaling system, also an issue for further investigations.

Further, our network analyses identified five modules where the largest sub-network was centered on PEX26 and comprised proteins involved in division and proliferation (PEX11A/B/G, FIS1, and DNM1L), matrix protein import (PEX5/6/7/10/12/13/14), and proteins representing both Zellweger spectrum diseases and peroxisomal division defects. These observations support the notion that PEX26 deficiency is related to dysfunction of other processes than matrix protein import and may additionally be associated to phenotypes arising from pathobiological processes of peroxisomal proliferation and division. The integrity of the peroxisomal protein interaction network relies on the high connectivity of PEX26 and, to a lesser extent, of PEX19, PEX5, and PEX7. The latter proteins are mainly cytosolic and responsible for import of membrane and matrix proteins in peroxisomal biogenesis. Node removal of PEX26 out of all proteins localized at the peroxisomal membrane or matrix would result in a complete breakdown of the network architecture. As a consequence, the degree of edgetic perturbations of PEX26 determines maintenance of network integrity and thus proper residual function of the whole peroxisome as well as of affected, shared cellular pathways. Upon node removal of PEX26, the three functional motifs associated with fatty acid metabolism, division and proliferation, and matrix protein import, respectively, were maintained but interconnection of the motifs was lost. In addition, loss of PEX26 interactions resulted in four isolated nodes associated with fatty acid metabolism. Peroxisomes are involved in different pathways of lipid metabolism that are on one hand interrelated with each other and on the other hand shared by different organelles. Functional impairment of proteins interacting with PEX26 by perturbations of these interactions could affect VLCFA transport across the peroxisomal membrane (ABCD1, ABCD3, ACBD5), β-oxidation of some unsaturated fatty acids (ECH1), long-chain fatty alcohol as well as sphingolipid degradation (ALDH3A2), and plasmalogen synthesis (FAR1) (Figure 6D).

In conclusion, functional analyses of PEX26 deficiency with respect to different pathways of peroxisomal biogenesis and metabolic function demonstrated essentiality of this protein. Given that PEX26 deficiency results in impaired function of proteins binding to PEX26, either due to node removal or edgetic perturbations, these pathways would be affected at different steps of both transport and turnover of their metabolites. The clinical phenotype of ZS in individuals carrying genotypes with nonsense variants or other types of loss-of-function variants could therefore not only evolve from a disrupted function of the PEX26-PEX6-PEX1 complex, but also from functional impairment of other processes linked by the PEX26 sub-network.

Our study showed that PEX26 establishes a functional link to fatty acid metabolism. The involvement of peroxisomes in cellular lipid metabolism may exemplify how pathways of nutrient catabolism, anabolism, and cell signaling are highly interrelated between different organelles providing metabolites for virtually all tissues. The pathways of fatty acid β-oxidation, docosahexaenoic acid metabolism, and plasmalogen synthesis all pass through the peroxisome and several components are shared between these processes. Given that PEX26 deficiency produces impaired function of proteins binding to PEX26, either due to node removal or edgetic perturbations, these pathways would be affected at different steps of both transport and turnover of their metabolites. This may add to the observation that VLCFA accumulation and plasmalogen biosynthesis are interrelated peroxisomal pathways in particular with respect to pathobiology of peroxisomal disorder (Brites et al., 2009), an issue for further investigations.

Fine mapping of the impact of individual variants on binary PEX26 interactions showed robust correlation of genotype and both molecular and clinical phenotypes. This analysis revealed specific patterns of edge losses and edge-loss distributions for single PEX26 variants. Based on these patterns, variants were grouped into three different clusters that correlated with PEX26 dysfunction. The number of affected interactions correlated with the molecular phenotype of matrix protein import, i.e., the more interactions were maintained for a specific variant, the better peroxisomal matrix proteins were imported into the peroxisomes. In addition, the number of affected interactions correlated with the number of phenotype gene relationships affected by edgetic perturbations, i.e., the severely affected cluster 3 was associated with the highest number of disease phenotypes. This notion might be regarded as self-evident, however, eight of 18 proteins interacting with PEX26 are not directly related to diseases. On the other hand, our observations underscore the significance of functional cellular networks for the development of the patient’s phenotype where the extent and nature of edgetic perturbations may significantly contribute to disease expression. This is exemplified with respect to *p*.Pro117Leu. The variant induced the highest number of edgetic perturbations, was associated with many phenotype gene relationships, and displayed a severe biochemical phenotype of disrupted matrix protein import. However, a patient compound heterozygote for *p*.Pro117Leu with *p*.Leu153Val+288fs in trans showed a mild clinical phenotype of IRD. This may be due to phenotype attenuation by the second allele. Given that *p*.Leu153Val was assigned to cluster 1 together with wild-type PEX26 and displayed the mildest forms of edgetic perturbations and biochemical phenotypes, it is likely that a patient homozygote for *p*.Pro117Leu would display a severe phenotype. This supports the hypothesis that extent and nature of edgetic perturbations may significantly contribute to disease expression.

The low frequency of missense variants causing PEX26 deficiency together with the limited number of different genotypes and the fact that most patients are compound heterozygotes impede to establish clear correlations of single variant with clinical phenotypes. Upon identification of more patients carrying homozygote or functional hemizygote genotypes of a robust correlation between variants and clinical phenotypes based on edgetic perturbations may be anticipated.

In spite of the numerous different metabolic pathways peroxisomes are involved in, peroxisomal disorders share, with few exceptions, the common feature of developing phenotypes of neurological impairment. Interestingly, they build a broad spectrum of different pathogenic entities comprising disorders of brain development, neurodegeneration, neurotoxicity, neurovasculation, and oncologic traits. These disorders affect a variety of cell types and organs of the central nervous system such as white matter, peripheral nerves, motoneurons, retina or cerebellum. This may lead to the hypothesis that virtually any impairment of the peroxisomal network impacts processes of fundamental importance for proper development and function of the nervous system. In particular, nature and degree of variant induced perturbations of protein interactions may govern the balance of (phospho)lipid metabolism.

The notion of interrelated peroxisomal processes of biogenesis and metabolism may also yield mechanistic insight into therapeutic intervention in peroxisomal biogenesis disorders, where pharmacological treatment with 4-phenylbutyrate concomitantly induced PEX11A dependent peroxisomal proliferation and ABCD2 mediated peroxisomal transport of fatty acids (Kemp et al., 1998; Wei et al., 2000). It is tempting to speculate that impaired function of PEX11B or ABCD1 in PEX26 deficiency may be partially rescued by increased expression of the functionally related proteins PEX11A and ABCD2. Moreover, this link extends to other diseases associated with peroxisomal dysfunction. In mice, the deletion of a single allele of *PEX11B*, involved in proliferation and fatty acid transport, was sufficient to cause oxidative stress and neuronal death (Ahlemeyer et al., 2012) Genome wide association studies revealed a relation of peroxisomal fatty oxidation genes with susceptibility for and treatment outcome of leukemia (Di Bernardo et al., 2008; Wade et al., 2011). In the context of the metabolic syndrome, diet induced hepatic steatosis stimulated peroxisomal fatty acid oxidation and increased generation of reactive oxygen species (Hall et al., 2010; Rolo et al., 2012), whereas efficient peroxisomal elimination of these agents prevented lipotoxicity in type 2 diabetes (Elsner et al., 2011). Recent research focused on the role of peroxisomal metabolism and peroxisomal dysfunction in the development or progression of Alzheimer’s disease and other neurodegenerative disorders (Kou et al., 2011; Lizard et al., 2012; Saez-Orellana et al., 2020; Zarrouk et al., 2020). This link mainly refers to alterations in docosahexaenoic acid (Astarita et al., 2010; Bazan et al., 2011) and plasmalogen (Goodenowe et al., 2007) levels, but an involvement of very long-chain fatty acid metabolism in these individuals is also discussed (Lizard et al., 2012), along with increased peroxisomal proliferation (Lizard et al., 2012) and lipid peroxidation (Arlt et al., 2002; Singh et al., 2010).

In summary, the PEX26 protein interaction network links different metabolic pathways and it takes a central hub position inside the peroxisomal interactome. Metabolic pathways passing through this network are associated with multigenic acquired diseases and PEX26 function might be of more significance for lipid metabolism and other major cellular processes than currently appreciated. Precise knowledge of functional aspects in a given network may therefore improve accuracy in network medicine strategies and detailed mapping of edgetic perturbations will set the basis for novel targets for specific therapeutic intervention.

## Data Availability

The original contributions presented in the study are included in the article/Supplementary Material, further inquiries can be directed to the corresponding authors.
