# Supplementary material for: Edgetic Perturbations Contribute to Phenotypic Variability in PEX26 Deficiency
Source: Front Genet. 2021 Nov 4;12:726174. doi: 10.3389/fgene.2021.726174 (PMC8600046; doi:10.3389/fgene.2021.726174)
Supplement: Supplementary file 1 [file DataSheet1.pdf]

Supplementary material

1. **SUPPLEMENTARY FIGURES**

Supplementary Figure 1 - This figure is related to Fig. 3*C* and demonstrates the results of BRET analysis as the basis for the determination of binding domains of PEX26 to the newly identified interaction partners.

2. **SUPPLEMENTARY TABLES**

Supplementary Table 1 - This table is related to the Results section *"Interaction of wild-type and variant PEX26 with PEX6" and "PEX26 variant-induced edgetic perturbations of the peroxisomal interactome"* of the main manuscript and provides additional information on genotypes and phenotypes of *PEX26* variants investigated in this study.

Supplementary Table 2 - This table is related to Fig. 2*A* and provides the list of genes encoding peroxisomal proteins investigated in this study.

Supplementary Table 3 – This table relates to Fig. 2*C* and provides results from OMIM database research and analysis.

Supplementary Table 4 - This table is related to Fig. 4*B* and demonstrates the matrix of PPI for WT and variant PEX26 used as a basis for cluster analysis.

Supplementary Table 5 - This table is related to Fig. 4*A* and provides parameters of network analysis.

Supplementary Table 6 - This table is related to Fig. 4*C* and provides additional information on biochemical phenotype parameters.

3. **SUPPLEMENTARY REFERENCES**

References cited in the supplemental Tables and Experimental Procedures section described above are given.

SUPPLEMENTARY FIGURES

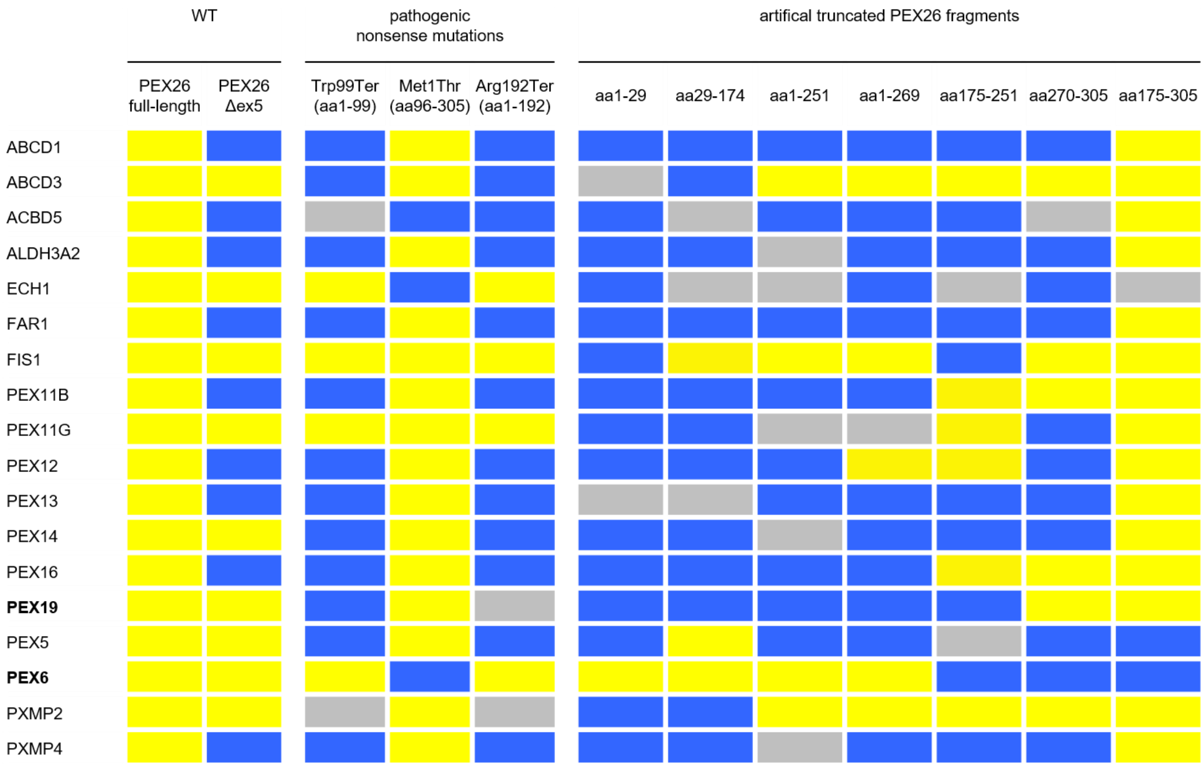

Supplementary Figure 1. Characterization of PEX26 binding domains to the newly identified interaction partner. Interaction matrix of full-length PEX26 and truncation constructs analyzed by BRET. Yellow nodes represent a positive interaction in at least one out of eight combinations tested, blue nodes represent negative interactions in all combinations, grey nodes represent those tested combinations that resulted in conflicting assertions.

SUPPLEMENTARY TABLES

Supplementary Table 1. Phenotypes and genotypes of *PEX26* variants investigated in this study.

Variant	Biochemical phenotype: PEX6 binding*	Biochemical phenotype: Peroxisomal matrix protein import†	Genotype‡	Clinical phenotype‡
p.Met1Thr	< 40%	55%	*p*.Met1Thr/*p*.Leu45Pro	IRD
p.Leu44Pro	40-70%	0%	*p*.Leu44Pro/*p*.Arg98Trp	NALD
p.Leu45Pro	40-70%	0%	*p*.Leu45Pro/*p*.Met1Thr	IRD
p.Gly89Arg	70-90%	0%	*p*.Gly89Arg/*p*.Gly89Arg	ZS
p.Arg98Trp	100%	20%	*p*.Arg98Trp/*p*.Arg98Trp	NALD
			p.Arg98Trp/*p*.Leu44Pro	NALD
			p.Arg98Trp/*p*.Ala143_Val182dup+ *p*.Gly183Val	IRD
p.Trp99Ter	no data available			
p.Pro117Leu	40-70%	0%	*p*.Pro117Leu/*p*.Leu153Val+ *p*.Arg288fs366Ter	IRD
p.Pro118Arg	no data available			
p.Leu153Val	100%	70%	*p*.Leu153Val+*p*.Arg288fs366Ter/ *p*.Pro117Leu	IRD
p.Arg192Ter	no data available			

Missense and nonsense variants in the *PEX26* gene investigated in this study and the related biochemical and clinical phenotypes. *PEX6 binding determined by co-immunpreciptiation (Furuki et al., 2006), †import of peroxisomal catalase at 37°C (Matsumoto et al., 2003). ‡Reported genotypes and clinical phenotypes associated with variants in *PEX26* (Matsumoto et al., 2003;Steinberg et al., 2004;Weller et al., 2005;Furuki et al., 2006). IRD, infantile Refsum disease; NALD, neonatal adrenoleukodystrophy; ZS, Zellweger syndrome.

Supplementary Table 2. Library of genes encoding peroxisomal proteins.

Gene ID	Isoform 1	Isoform 2
ABCD1	BC015541.1	
ABCD2	BC104903.1	
ABCD3	BC009712.2	
ABCD4	BC012815.2	
ACAA1	BC011977.1	BC014474.1
ACAD11	BC125204.1	
ACBD5	BC030555.1	
ACOT1	BC132891.1	
ACOT2	BC006335.1	AY005822.1
ACOT4	BC117343.1	
ACOX1	BC010425.1	
ACOX2	BC047700.1	
ACOX3	BC017053.1	
ACSF3	BC072391.1	
ACSL4	BC034959.2	
ACSL5	BC007985.2	
ACSL6	BC047453.1	
AGPS	BC141820.1	
AGXT	BC132819.1	
ALDH3A2	BC002430.2	
AMACR	BC009471.1	
BAAT	BC009567.1	
CAT	BC112217.1	
CRAT	BC000723.2	
CROT	BC039004.1	
DAO	BC029057.1	
DDO	BC032786.1	
DECR2	BC010740.1	
DHRS4	BC003019.1	
DNAJC10	BC117299.1	
DNM1L	BC024590.1	

ECH1	BC011792.2	
EHHADH	BC038948.1	
EPHX2	BC007708.2	
FAR1	BC017377.2	
FAR2	BC022267.1	
FIS1	BC003540.1	
FNDC5	BC062297.1	
GNPAT	BC000450.2	
GSTK1	AL136938.1	BC063425.1
HACL1	BC001627.1	
HAO1	BC113665.1	
HAO2	BC020863.1	
HMGCL	BC010570.1	
HSD17B4	BC003098.1	
IDH1	BC012846.1	
IDI1	BC019227.2	
IDI2	BC017778.1	
MFF	BC093024.1	
MLYCD	BC052592.1	
MOSC2	BC011973.1	
MPV17	BC001115.2	
MPV17L2	BC005064.1	
MVK	BC016140.1	
NOS2	BC130283.1	
NUDT12	BC041099.1	
PECI	BC002668.2	
PEX1	BC035575.1	
PEX10	BC000543.2	NM_002617.3
PEX11A	BC009697.1	
PEX11B	BC011963.1	
PEX11G	BC008780.2	
PEX12	BC031085.1	
PEX13	BC067090.1	
PEX14	BC006327.2	
PEX16	BC004356.1	
PEX19	DQ894591.2	
PEX2	BC005375.1	
PEX26	NM_001127649.1	
PEX3	BC015506.1	
PEX5	NM_001131025.1	
PEX6	BC048331.1	
PEX7	BC031606.1	
PHYH	BC029512.1	
PIPOX	BC008960.2	
PMVK	BC006089.1	
PRDX1	BC007063.1	
PRDX5	BC113725.1	
PXMP2	BC073997.1	
PXMP4	BC001147.1	
PXT1	BC107049.2	
RHOC	BC007245.1	
SCP2	BC005911.1	BC067108.1
SLC22A5	BC012325.1	
SLC25A17	BC005957.1	
SLC27A2	BC057770.1	
SOD1	BC001034.1	
SOD2	BC012423.1	BC016934.1
TMEM135	BC051462.1	
TRIM37	BC036012.1	
ZADH2	BC033780.1	

Peroxisomal protein library covering 88% of all proteins annotated with peroxisomal localization. All proteins were analyzed for interaction with PEX26 in a BRET-based PPI-screen. For 6 out of the 90 proteins, 2 isoforms were analyzed.

Supplementary Table 3. Results from OMIM database research and analysis.

Source	Target	Type	OMIM	Alternate Name
ACOX1	ACOXX	dg	264470	PEROXISOMAL ACYL-CoA OXIDASE DEFICIENCY
ABCD1	ALD	dg	300100	ADRENOLEUKODYSTROPHY
AMACR	AMACRD	dg	614307	ALPHA-METHYLACYL-CoA RACEMASE DEFICIENCY
PEX7	ARD1	dg	614879	REFSUM DISEASE, ADULT, 2
PHYH	ARD2	dg	266500	REFSUM DISEASE, ADULT, 1

AMACR	CBAS4	dg	214950	BILE ACID SYNTHESIS DEFECT, CONGENITAL, 4
NSDHL	CHILDS	dg	308050	CONGENITAL HEMIDYSPLASIA WITH ICHTHYOSIFORM ERYTHRODERMA AND LIMB DEFECTS
HSD17B4	DBPX	dg	261515	PEROXISOMAL BIFUNCTIONAL ENZYME DEFICIENCY
DNM1L	EMPF	dg	614388	ENCEPHALOPATHY, LETHAL, DUE TO DEFECTIVE MITOCHONDRIAL AND PEROXISOMAL FISSION
C7orf10	GA3	dg	231690	GLUTARIC ACIDURIA III
AGXT	HP1	dg	259900	HYPEROXALURIA, PRIMARY, TYPE I
GUCY2D	LCA1	dg	204000	LEBER CONGENITAL AMAUROSIS 1
MLYCD	MCDX	dg	248360	MALONYL-CoA DECARBOXYLASE DEFICIENCY
TRIM37	MN	dg	253250	MULIBREY NANISM
PEX1	IRD	dg	601539	INFANTILE REFSUM DISEASE
PEX12	IRD	dg	266510	INFANTILE REFSUM DISEASE
PEX16	IRD	dg	614877	INFANTILE REFSUM DISEASE
PEX26	IRD	dg	614873	INFANTILE REFSUM DISEASE
PEX5	IRD	dg	202370	INFANTILE REFSUM DISEASE
PEX6	IRD	dg	614863	INFANTILE REFSUM DISEASE
PXMP3	IRD	dg	614867	INFANTILE REFSUM DISEASE
PEX1	NALD	dg	601539	NEONATAL ADRENOLEUKODYSTROPHY
PEX10	NALD	dg	614871	NEONATAL ADRENOLEUKODYSTROPHY
PEX12	NALD	dg	266510	NEONATAL ADRENOLEUKODYSTROPHY
PEX13	NALD	dg	614885	NEONATAL ADRENOLEUKODYSTROPHY
PEX16	NALD	dg	614877	NEONATAL ADRENOLEUKODYSTROPHY
PEX26	NALD	dg	614873	NEONATAL ADRENOLEUKODYSTROPHY
PEX5	NALD	dg	202370	NEONATAL ADRENOLEUKODYSTROPHY
PEX6	NALD	dg	614863	NEONATAL ADRENOLEUKODYSTROPHY
PXMP3	NALD	dg	614867	NEONATAL ADRENOLEUKODYSTROPHY
NPC1	NPC	dg	257220	NIEMANN-PICK DISEASE, TYPE C1
PEX11B	PDX	dg	614920	PEROXISOME DIVISION DEFICIENCY
HSD17B4	PRLTS1	dg	233400	PERRAULT SYNDROME 1
PEX7	RCDP1	dg	215100	RHIZOMELIC CHONDRODYSPLASIA PUNCTATA, TYPE 1
GNPAT	RCDP2	dg	222765	RHIZOMELIC CHONDRODYSPLASIA PUNCTATA, TYPE 2
AGPS	RCDP3	dg	600121	RHIZOMELIC CHONDRODYSPLASIA PUNCTATA, TYPE 3
SCP2	SCPX	dg	613724	LEUKOENCEPHALOPATHY WITH DYSTONIA AND MOTOR NEUROPATHY
PEX1	ZS	dg	214100	ZELLWEGER SYNDROME
PEX10	ZS	dg	614870	ZELLWEGER SYNDROME
PEX12	ZS	dg	614859	ZELLWEGER SYNDROME
PEX13	ZS	dg	614883	ZELLWEGER SYNDROME
PEX14	ZS	dg	614887	ZELLWEGER SYNDROME
PEX16	ZS	dg	614876	ZELLWEGER SYNDROME
PEX19	ZS	dg	614886	ZELLWEGER SYNDROME
PEX26	ZS	dg	614872	ZELLWEGER SYNDROME
PEX3	ZS	dg	614882	ZELLWEGER SYNDROME
PEX5	ZS	dg	214110	ZELLWEGER SYNDROME
PEX6	ZS	dg	614862	ZELLWEGER SYNDROME
PXMP3	ZS	dg	614866	ZELLWEGER SYNDROME
ALDH3A2	SLS	dg	270200	SJOGREN-LARSSON SYNDROME
MPV17	MTDPS6	dg	256810	MITOCHONDRIAL DNA DEPLETION SYNDROME 6
ERBB2	GLM1	dg	137800	GLIOMA SUSCEPTIBILITY 1
HMGCL	HMGCLD	dg	246450	3-HYDROXY-3-METHYLGLUTARYL-CoA LYASE DEFICIENCY
ACSL4	MRX63	dg	300387	MENTAL RETARDATION, X-LINKED 63
MVK	HIDS	dg	260920	HYPER-IgD SYNDROME
MVK	POROK3	dg	175900	POROKERATOSIS 3, DISSEMINATED SUPERFICIAL ACTINIC TYPE
MVK	MEVA	dg	610377	MEVALONIC ACIDURIA
ACSF3	CMAMMA	dg	614265	COMBINED MALONIC AND METHYLMALONIC ACIDURIA
BAAT	FHCA	dg	607748	HYPERCHOLANEMIA, FAMILIAL
SLC22A5	CDSP	dg	212140	CARNITINE DEFICIENCY, SYSTEMIC PRIMARY
ABCD4	MAHCJ	dg	614857	METHYLMALONIC ACIDURIA AND HOMOCYSTINURIA, cblJ TYPE
EPHX2	FHC	dg	143890	HYPERCHOLESTEROLEMIA, FAMILIAL
DNM1L	EMPF	dg	614388	DEFECTIVE MITOCHONDRIAL AND PEROXISOMAL FISSION
DAO	SCZD	dg	181500	SCHIZOPHRENIA
CAT	CATD	dg	115500	ACATALASEMIA
AMACRD	CBAS4	dd		
RCDP1	ARD1	dd		
DBPX	PRLTS1	dd		
NALD	ZS	dd		

IRD	ZS	dd
NALD	IRD	dd
POROK3	MEVA	dd
HIDS	MEVA	dd
POROK3	HIDS	dd
ABCD1	ABCD1	pp
ABCD1	ABCD2	pp
ABCD1	ABCD3	pp
ABCD1	PEX19	pp
ABCD1	PEX26	pp
ABCD2	ABCD2	pp
ABCD2	ABCD3	pp
ABCD2	PEX19	pp
ABCD3	ABCD3	pp
ABCD3	PEX19	pp
ABCD3	PEX26	pp
ACBD5	PEX26	pp
ACOX1	SCP2	pp
ACSL6	PHYH	pp
AGPS	GNPAT	pp
AGXT	PEX5	pp
ALDH3A2	PEX26	pp
ASNA1	ASNA1	pp
CAT	CAT	pp
CAT	PEX5	pp
DAO	DAO	pp
DDO	PEX5	pp
DECR2	PEX19	pp
DNM1L	FIS1	pp
DNM1L	PEX11A	pp
DNM1L	PEX11B	pp
DNM1L	PEX11G	pp
ECH1	PEX26	pp
EHHADH<br>2	SCP2	pp
FAR1	PEX26	pp
FIS1	FIS1	pp
FIS1	PEX11A	pp
FIS1	PEX11B	pp
FIS1	PEX11G	pp
FIS1	PEX26	pp
HACL1	HACL1	pp
MVK	MVK	pp
PEX1	PEX6	pp
PEX10	PEX10	pp
PEX10	PEX12	pp
PEX10	PEX19	pp
PEX10	PEX5	pp
PEX10	PXMP3	pp
PEX11A	PEX11A	pp
PEX11A	PEX11G	pp
PEX11A	PEX19	pp
PEX11B	PEX11B	pp
PEX11B	PEX11G	pp
PEX11B	PEX19	pp
PEX11B	PEX26	pp
PEX11G	PEX11G	pp
PEX11G	PEX26	pp
PEX12	PEX19	pp
PEX12	PEX26	pp
PEX12	PEX5	pp
PEX13	PEX13	pp
PEX13	PEX14	pp
PEX13	PEX19	pp
PEX13	PEX26	pp
PEX13	PEX5	pp
PEX13	PEX7	pp

PEX14	PEX14	pp
PEX14	PEX19	pp
PEX14	PEX26	pp
PEX14	PEX5	pp
PEX14	PEX7	pp
PEX16	PEX19	pp
PEX16	PEX26	pp
PEX19	PEX26	pp
PEX19	PEX3	pp
PEX19	PXMP4	pp
PEX19	SLC25A17	pp
PEX26	PEX11B	pp
PEX5	PEX26	pp
PEX5	PEX7	pp
PEX5	SCP2	pp
PEX6	PEX26	pp
PEX7	PHYH	pp
PHYH	PHYH	pp
PRDX1	PRDX1	pp
PXMP2	PEX26	pp
PXMP4	PEX26	pp
SOD1	SOD1	pp
SOD1	SOD2	pp
TRIM37	TRIM37	pp
AMACRD	CBAS4	dd
RCDP1	ARD1	dd
DBPX	PRLTS1	dd
NALD	ZS	dd
IRD	ZS	dd
NALD	IRD	dd
POROK3	MEVA	dd
HIDS	MEVA	dd
POROK3	HIDS	dd

Supplementary Table 4. Binary PPI matrix of edgetic perturbations.

	WT	*p*.Leu153Val	*p*.Leu44Pro	*p*.Leu45Pro	*p*.Pro118Arg	*p*.Gly89Arg	*p*.Arg98Trp	*p*.Pro117Leu
ABCD1	1	1	1	1	0	0	0	0
ABCD3	1	0	0	0	0	0	0	0
ACBD5	1	0	0	0	0	0	0	0
ALDH3A2	1	1	1	1	1	0	0	1
ECH1	1	1	1	1	1	1	1	1
FAR1	1	1	0	0	0	0	0	0
FIS	1	1	1	1	1	1	1	1
PEX11B	1	1	0	0	0	0	0	0
PEX11G	1	1	1	1	1	1	1	0
PEX12	1	1	1	1	1	1	1	0
PEX13	1	1	0	1	0	0	0	0
PEX14	1	1	1	1	1	1	1	1
PEX16	1	0	0	0	0	0	0	0
PEX19	1	1	1	1	1	1	1	1
PEX5	1	1	1	1	1	1	1	1
PEX6	1	1	0	0	0	0	0	0
PXMP2	1	1	1	1	1	1	1	1
PXMP4	1	0	1	1	1	1	1	1

Matrix of PPI for WT and variant PEX26 with proteins interacting with WT PEX26. Positive interactions were classified as *1*, non-interacting was classified as *0*.

Supplementary Table 5. Network analysis of the PEX26 associated peroxisomal interactom.

	Clustering coefficient	Connected components	Network centrality	Average number of neighbours	Number of nodes	Network density	Network heterogeneity	Isolated nodes	Number of edges
WT	0.273	57	0.429	3.405	37	0.095	1.087	0	74
p.Leu153Val	0.215	58	0.347	3.189	37	0.089	1.054	1	70
p.Leu44Pro	0.229	60	0.352	3.027	37	0.084	1.032	2	67
p.Leu45Pro	0.234	60	0.350	3.081	37	0.086	1.041	2	68
p.Pro118Arg	0.229	60	0.353	2.973	37	0.083	1.027	2	66
p.Gly89Arg	0.230	61	0.355	2.919	37	0.081	1.034	3	65
p.Arg98Trp	0.230	61	0.355	2.919	37	0.081	1.034	3	65
p.Pro117Leu	0.231	60	0.356	2.865	37	0.08	1.021	2	64
p.Met1Thr	0.274	60	0.345	3.243	37	0.09	1.067	2	71
p.Trp99Ter	0.203	61	0.333	2.649	37	0.074	1.006	4	60
p.Arg192Ter	0.203	61	0.333	2.649	37	0.074	1.006	4	60
Node removal	0.217	63	0.348	2.500	36	0.071	1.073	5	56

Parameters of network analysis are given for the peroxisomal network comprising PPI identified in this study merged with a dataset of known peroxisomal PPI that are directly or indirectly associated to the PEX26 network. Data are given for the network in dependence of variants in PEX26.

Supplementary Table 6. Correlation of edgetic pertubations to phenotypic parameters.

		Number of PPI	Protein amount	Matrix protein import (37°C)			Biochemical score
				Catalase	PTS1	PTS2	
WT		18	30	10	10	10	60
p.Leu153Val		14	15	7	8	10	40
p.Leu44Pro		12	30	0	0	0	30
p.Leu45Pro		12	30	0	0	0	30
p.Pro118Arg		10	n.a.	n.a.	n.a.	n.a.	n.a.
p.Gly89Arg		10	15	0	0.5	0	15.5
p.Arg98Trp		10	15	2	7	7	31
p.Pro117Leu		9	15	0	2	1	18
Correlation to number of PPI	Pearson r		0.56	0.89	0.65	0.67	0.95
	P-value		0.187	0.008	0.115	0.102	0.001

Number of protein-protein interactions (PPI) derived from BRET experiments are depicted for each variant and WT PEX26. The biochemical score is weighted 30-10-10-10-10 with respect to protein amount (Furuki et al., 2006), catalase import, PTS1-dependent import, and PTS2 dependent import to yield a total of 60 for WT PEX26. For the protein amount, a value of 30 reflects a protein stability comparable to the WT PEX26 and a score of 15 reflects reduced but residual protein stability. For peroxisomal matrix protein import (Matsumoto et al., 2003) a score of 10 corresponds to 100% of matrix protein import.

SUPPLEMENTARY REFERENCES

Furuki, S., Tamura, S., Matsumoto, N., Miyata, N., Moser, A., Moser, H.W., et al. (2006). Mutations in the peroxin Pex26p responsible for peroxisome biogenesis disorders of complementation group 8 impair its stability, peroxisomal localization, and interaction with the Pex1p x Pex6p complex. *J Biol Chem* 281**,** 1317-1323.

Gersting, S.W., Lotz-Havla, A.S., and Muntau, A.C. (2012). Bioluminescence resonance energy transfer: an emerging tool for the detection of protein-protein interaction in living cells. *Methods Mol Biol* 815**,** 253-263.

Hamdan, F.F., Percherancier, Y., Breton, B., and Bouvier, M. (2006). Monitoring protein-protein interactions in living cells by bioluminescence resonance energy transfer (BRET). *Curr Protoc Neurosci* Chapter 5**,** Unit 5 23.

Hillebrand, M., Gersting, S.W., Lotz-Havla, A.S., Schaefer, A., Rosewich, H., Valerius, O., et al. (2011). Identification of a new fatty acid synthesis-transport machinery at the peroxisomal membrane. *J Biol Chem.*

Matsumoto, N., Tamura, S., Furuki, S., Miyata, N., Moser, A., Shimozawa, N., et al. (2003). Mutations in novel peroxin gene PEX26 that cause peroxisome-biogenesis disorders of complementation group 8 provide a genotype-phenotype correlation. *Am J Hum Genet* 73**,** 233-246.

Smoot, M.E., Ono, K., Ruscheinski, J., Wang, P.L., and Ideker, T. (2011). Cytoscape 2.8: new features for data integration and network visualization. *Bioinformatics* 27**,** 431-432.

Steinberg, S., Chen, L., Wei, L., Moser, A., Moser, H., Cutting, G., et al. (2004). The PEX Gene Screen: molecular diagnosis of peroxisome biogenesis disorders in the Zellweger syndrome spectrum. *Mol Genet Metab* 83**,** 252-263.

Weller, S., Cajigas, I., Morrell, J., Obie, C., Steel, G., Gould, S.J., et al. (2005). Alternative splicing suggests extended function of PEX26 in peroxisome biogenesis. *Am J Hum Genet* 76**,** 987-1007.
